# Inhibition of Cell Proliferation and Cell Viability by Sinecatechins in Cutaneous SCC Cells Is Related to an Imbalance of ROS and Loss of Mitochondrial Membrane Potential

**DOI:** 10.3390/antiox11071416

**Published:** 2022-07-21

**Authors:** Jiaqi Zhu, Bernd Gillissen, Dieu Linh Dang Tran, Stefanie May, Claas Ulrich, Eggert Stockfleth, Jürgen Eberle

**Affiliations:** 1 Skin Cancer Centre Charité, Department of Dermatology and Allergy, Charité–Universitätsmedizin Berlin, Charitéplatz 1, 10117 Berlin, Germany; jiaqi.zhu@charite.de (J.Z.); tran-dieu-linh.dang@charite.de (D.L.D.T.); stefanie.may@charite.de (S.M.); claas.ulrich@charite.de (C.U.); 2 Department of Gynecology and Obstetrics, Jilin University, Changchun 130001, China; 3 Department of Hematology, Oncology, and Tumor Immunology, Charité–Universitätsmedizin Berlin, 13125 Berlin, Germany; bernhard.gillissen@charite.de; 4 Beuth-Hochschule für Technik Berlin–University of Applied Sciences, Luxemburger Str. 10, 13353 Berlin, Germany; 5 Dermatologie, Venerologie und Allergologie, Klinikum Bochum, Ruhr-Universität Bochum, Gudrunstr. 56, 44791 Bochum, Germany; e.stockfleth@klinikum-bochum.de

**Keywords:** cancer, therapy, cell viability, reactive oxygen species, apoptosis

## Abstract

The term sinecatechins designates an extract containing a high percentage of catechins obtained from green tea, which is commercially registered as Veregen or Polyphenon E (PE) and may be considered for treatment of cutaneous squamous cell carcinoma (cSCC) and actinic keratosis (AK). As shown here, treatment of four cSCC cell lines with 200 µg/mL of PE resulted in strong, dose-dependent decrease in cell proliferation (20–30%) as well as strongly decreased cell viability (4–21% of controls, 48 h). Effects correlated with loss of mitochondrial membrane potential, whereas early apoptosis was less pronounced. At the protein level, some activation of caspase-3 and enhanced expression of the CDK inhibitor p21 were found. Loss of MMP and induced cell death were, however, largely independent of caspases and of the proapoptotic Bcl-2 proteins Bax and Bak, suggesting that sinecatechins induce also non-apoptotic, alternative cell death pathways, in addition to apoptosis. Reactive oxygen species (ROS) were downregulated in response to PE at 4 h, followed by an increase at 24 h. The contributory role of initially reduced ROS was supported by the antioxidant N-acetyl cysteine, which in combination with PE further enhanced the negative effects on cell viability. Thus, sinecatechins inhibited cell proliferation and viability of cSCC cells, which could suggest the use of PE for AK treatment. The mechanisms appear as linked to an imbalance of ROS levels.

## 1. Introduction

About 20% of skin malignancies and skin cancer deaths worldwide result from cutaneous squamous cell carcinoma (cSCC) [1,2]. Thus, following basal cell carcinoma, cSCC is the second most common skin cancer in Caucasians and East Asians [3,4]. Actinic keratosis (AK) is characterized by a particularly high prevalence and is mainly located on sun-exposed areas of the skin. Clinical and subclinical lesions often coexist across a large area described as field cancerization [5]. As AK derives from neoplastic epidermal keratinocytes, and as lesions have the potential to transform into invasive cSCC, AK was defined as cSCC in situ, and its treatment is crucial [6].

Several therapies have been established for AK/cSCC in recent years, and the green tea extract sinecatechins (PE) represent a promising candidate [7,8]. PE contains a particularly high proportion of epigallocatechin-3-gallate (EGCG; 55%) as well as other polyphenolic compounds as epigallocatechin, epicatechin-3-gallate, epicatechin, gallocatechins, and gallocatechin gallate [9]. For EGCG, inhibition of cell proliferation and induction of apoptosis have already been reported in mammary carcinoma, lung cancer and gastric cancer cells [10,11,12].

Cell proliferation is critically regulated by cyclin-dependent kinases (CDK 1/2/4/6), which mediate retinoblastoma (Rb) protein phosphorylation, thus resulting in E2F transcription factor-induced gene expression and activation of the cell cycle [13]. On the other hand, a panel of CDK inhibitors as of the INK4 family (p15, p16, p18, and p19) as well as of the Cip/Kip family (p21, p27 and p57) can prevent cell cycle progression [14]. While CDK inhibitors critically control cell proliferation in normal cells, they may be downregulated or abolished in cancer [15].

Induction of apoptosis represents an important goal in cancer therapy [16], and intrinsic proapoptotic pathways are activated by different anticancer drugs. This relies on mitochondrial membrane permeability and loss of mitochondrial membrane potential, which may further result in caspase activation [17]. The major effector caspase (caspase-3) cleaves multiple death substrates, which finally results in DNA fragmentation and apoptosis induction. Caspase-3 itself can be activated by the initiator caspase of the extrinsic apoptosis pathway (caspase-8) or by the initiator caspase of the intrinsic apoptosis pathway (caspase-9) [18]. The intrinsic, mitochondrial apoptotic pathways are mainly controlled by pro- and antiapoptotic Bcl-2 proteins. Here, the functionally related, proapoptotic, multidomain proteins Bax and Bak play essential roles, because, once activated, they cause mitochondrial outer membrane permeability and release of proapoptotic components from the intermembrane space into the cytosol [17]. In particular, mitochondria-mediated apoptosis is prevented in Bax/Bak knockout cells [19,20,21].

Reactive oxygen species are involved in different signaling pathways and play important roles in disease of the neuronal, cardiovascular, and nervous systems as well as in aging [22]. ROS have been further related to apoptosis induction in cancer cells, as recently shown in cSCC cells for celecoxib [23] and for indirubin derivatives [24].

Besides the often discussed possible roles of sinecatechins in cancer prevention, they were also suggested as therapeutic strategy for skin cancer as for AK [7]. Nevertheless, there is still only limited information on its direct effects and mechanisms in skin cancer cells. We therefore investigated its effects in four cSCC cell lines, which also represent cell culture models of AK. In particular, inhibition of cell proliferation, loss of cell viability, induction of apoptosis and molecular mechanisms were addressed.

## 2. Materials and Methods

### 2.1. Cells and Treatment

The effects of sinecatechins were studied in four established cSCC cell lines: SCL-I, SCL-II, SCC-12, and SCC-13. As AK is defined as cSCC in situ, these cell lines also serve as models for AK. SCC-12 and SCC-13 were kindly provided by Prof. J.G. Rheinwald (Department of Dermatology, Brigham and Women’s Hospital, Harvard Medical School, Boston, MA, USA); SCL-I and SCL-II were kindly provided by Prof. N.E. Fusenig (DKFZ, Heidelberg, Germany). SCC-12 and SCC-13 derive from rapidly growing, well-differentiated SCCs of the epidermis; SCL-I and SCL-II derive from squamous cell carcinomas of human skin. All four cell lines form monolayers in cell culture; atypical and poor stratification of colonies had been reported as well as tumorgenicity in nude mice [25,26]. SCL-I has shown resistance to diclofenac in a previous project, while the other cell lines were sensitive [27]. SCC cells were cultured at 5% CO_2_ in growth medium RPMI 1640 (Life Technologies, Darmstadt, Germany), which was supplemented with FCS (10%), glutamine (2 mM), non-essential amino acids and antibiotics.

For determination of Bax/Bak dependency, HCT-116 colon carcinoma wild-type cells (HCT-116 WT) and the isogenic double knockout subline HCT-116-Bax^−/−^/Bak^−/−^ (HCT-116 KO) were used, which had been kindly provided by Dr. R.J. Youle, National Institutes of Health, Bethesda, MD, USA [19]. HCT-116 cells were grown at 37 °C and 5% CO_2_ in DMEM growth medium (Invitrogen (Karlsruhe, Germany) supplemented with 10% FCS, penicillin and streptomycin. For control, normal human fibroblast cells (MRC-5) were used, which were derived from embryonic lung tissue (CCL-171, ATCC, Manassas, VA, USA).

Most assays were performed in 24-well plates, and 5 × 10^4^ cells were seeded per well. Sinecatechins (PE) was supplied by CPM Aenova (Feldkirchen, Germany); stock solutions were prepared in aqua iniectabilia at concentrations of 5 mg/mL, sterilized by filtration (0.2 µm filters) and stored at −80 °C. Light protection was applied at all steps. PE was applied in concentrations of 10, 25, 50, 100, and 200 µg/mL. For positive control, cells were treated with EGCG (Epigallocatechin gallate; Sigma-Aldrich E4143; 25 and 100 µg/mL). For further apoptosis control, cells were treated with an indirubin derivative (DKP-071, 10 µM) and/or TRAIL (TNF-related apoptosis inducing ligand; KillerTRAIL, Adipogen, San Diego, CA, AG-40T-0001; 50 ng/mL). The pan-caspase inhibitor Q-VD-OPH (Merck, Darmstadt, Germany, 5 µM) was applied 1 h before the other treatments.

### 2.2. Cell Proliferation

Relative cell proliferation rates were determined by WST-1 assay (Roche Diagnostics, Penzberg, Germany), which is based on staining cells with the water-soluble tetrazolium salt WST-1. In metabolically active cells, WST-1 is converted to formazan dye by mitochondrial dehydrogenases. The assay was quantified in an ELISA reader at 450 nm. As the enzyme activity is restricted to viable cells, the read-out reflects both cell numbers and cell viability. Thus, reduced WST-1 values may reflect either less cells (reduced cell proliferation) or less mitochondrial enzyme in single cells (less viable).

### 2.3. Apoptosis Induction and Cell Viability

For quantification of apoptosis by cell cycle analysis, cells were harvested by trypsinization and were dissolved in a hypotonic buffer containing sodium citrate (0.1%), triton-x 100 (0.1%) and propidium iodide (PI, Sigma-Aldrich, St. Louis, MO, USA, 40 µg/mL), in PBS. In this way, cells were lysed, and isolated cell nuclei were stained for at least 1 h with propidium iodide at 4 °C. Nuclei in cell cycle phases G1, G2 and S-phase as well as apoptotic sub-G1 nuclei were identified by flow cytometry at FL3A using a FACS Calibur (BD Bioscience, Bedford, MA, USA). As small DNA fragments are washed out of the nuclei by this procedure, apoptotic cells with DNA fragmentation are characterized by nuclei with less DNA (sub-G1 populations).

As an independent cell death assay, cells were stained with Annexin V-fluorescein isothiocyanate (AnnV) and propidium iodide (PI). In brief, cells harvested by trypsin/EDTA were washed twice with cold PBS and resuspended at 1 × 10^6^ cells/mL in 10 mM Hepes (pH 7.4), 140 mM NaCl, 2.5 mM CaCl_2_. For 100 µL of cell suspension (1 × 10^5^ cells), 5 µL of AnnV-FITC (BD Biosciences, Heidelberg, Germany) and 10 µL PI (20 mg/mL, Sigma-Aldrich) were added, and cells were incubated for 10 min on ice. Subsequently, samples were analyzed using a FACScan and CELLQuest software (Becton Dickinson, Heidelberg, Germany).

For determination of cell viability, cells were stained with calcein-AM (PromoCell, Heidelberg, Germany), which is transported through the cellular membrane into live cells. Upon transport, cellular esterases cut off the AM groups, the molecules bind to calcium within cells resulting in acquiring strong green fluorescence. As dead cells largely lack esterases, only live cells are efficiently marked. Cells were grown and treated in 24-well plates; they were harvested by trypsinization and stained with calcein-AM (0.5 µM) at 37 °C for 1 h. Before measurement by flow cytometry (FL2H), cells were washed two-times with PBS. Due to the evaluation by flow cytometry, the percentage of viable and non-viable cells can be determined, independently of the total cell number. As controls in calcein assays, cells without calcein labelling were also compared, and the effect of PE treatment on cell size was determined by monitoring FSC (forward scatter) in flow cytometry.

### 2.4. Mitochondrial Membrane Potential

For determination of the loss of mitochondrial membrane potential, cells were stained with the fluorescent dye tetramethylrhodamine-6-maleimide (TMRM^+^; Sigma-Aldrich). TMRM^+^ is transported into mitochondria with sufficient high mitochondrial membrane potential. Cells were grown and treated in 24-well plates; they were harvested by trypsinization and stained by TMRM^+^ (1 µM, 20 min, 37 °C). After 2× washing with PBS, cells were measured by flow cytometry (FL2H).

For microscopic visualization of MMP as well as of morphological changes in course of apoptosis, SCC cells were seeded into 6-well plates (2 × 10^5^ cells/2 mL) and treated for 24 h. Thereafter, cells were incubated for 30 min with 2 µg/mL JC-1 (5,5′,6,6′-tetrachloro-1,1′,3,3′-tetraethyl-benzimidazolylcarbocyanin iodide; Life Technologies) and with 0.2 µg/mL Hoechst-33342 (Sigma-Aldrich Chemie, Taufkirchen, Germany). After staining, microscopy images were taken with an Axiovert 200 inverse fluorescence microscope (Carl Zeiss, Jena, Germany) equipped with appropriate fluorescence filter sets and a Hamamatsu ORCA-ER digital camera.

### 2.5. Analysis of Reactive Oxygen Species

To determine intracellular ROS levels, cells were stained with the cell-permeable and non-fluorescent chemical 2’,7’-dichlorodihydrofluorescein diacetate (H_2_DCF-DA; D-399, Thermo Fisher Scientific, Hennigsdorf, Germany), which is oxidized in cells with high ROS levels to the fluorescent 2’,7’-dichlorodihydrofluorescein (DCF). Cells were grown in 24-well plates and were pre-incubated for 1 h with H_2_DCF-DA (10 µM), before starting treatment with other effectors. After 4–24 h of treatment, cells were harvested by trypsinization, washed two-times with PBS and were then analyzed by flow cytometry (FL1H). As positive controls, cells were treated with 1 mM H_2_O_2_ for 1 h. The antioxidant N-acetylcysteine (NAC, Sigma-Aldrich, Taufkirchen, Germany; 1 mM) was applied simultaneously with PE, aiming at a further decrease in PE-reduced ROS levels.

### 2.6. Western Blotting

For Western blotting, total proteins were extracted by lysing the cells in cell lysis buffer, containing 150 mM NaCl, 1 mM EDTA, 1% NP-40, 50 mM Tris-HCl, pH 8.0, and inhibitors for proteases and phosphatases. After SDS polyacrylamide gel electrophoresis, proteins were transferred by electro blotting to nitrocellulose membranes.

The following primary antibodies were used: Cleaved caspase-3 (9664, rabbit, 1:1000; Cell Signaling, Danvers, MA, USA); Caspase-8 (9746, mouse, 1:1000; Cell Signaling), Caspase-9 (9502, rabbit, 1:1000; Cell Signaling); p21 (sc-6246, mouse, 1:200; Santa Cruz Biotech, Dallas, TX, USA); p19 (sc-1063, rabbit, 1:200; Santa Cruz Biotech); β-actin (sc-47778, mouse, 1:200; Santa Cruz Biotech). The following secondary antibodies were used: peroxidase-labeled goat anti-rabbit and goat anti-mouse (Dako, Hamburg, Germany; 1:5000).

### 2.7. Statistical Analyses

Results of assays were generally proven by two to three independent experiments. Each individual experiment was consisted of triplicate values (three wells that were seeded, treated and analyzed individually). Thus for statistical analysis, there were at least six values in each group. For calculation of statistical significance, Student’s t-test was used, and significance is indicated by asterisks in all figures (* *p* < 0.05). Western blot experiments were repeated at least once by using two independent series of protein extracts. For semi-quantitative analysis, selected protein bands were quantified by densitometry, normalized by the respective β-actin signals, and median values were formed from two independent experiments.

## 3. Results

### 3.1. Dose-Dependent Inhibition of Cell Proliferation by Sinecatechins (PE)

**Figure 1:**
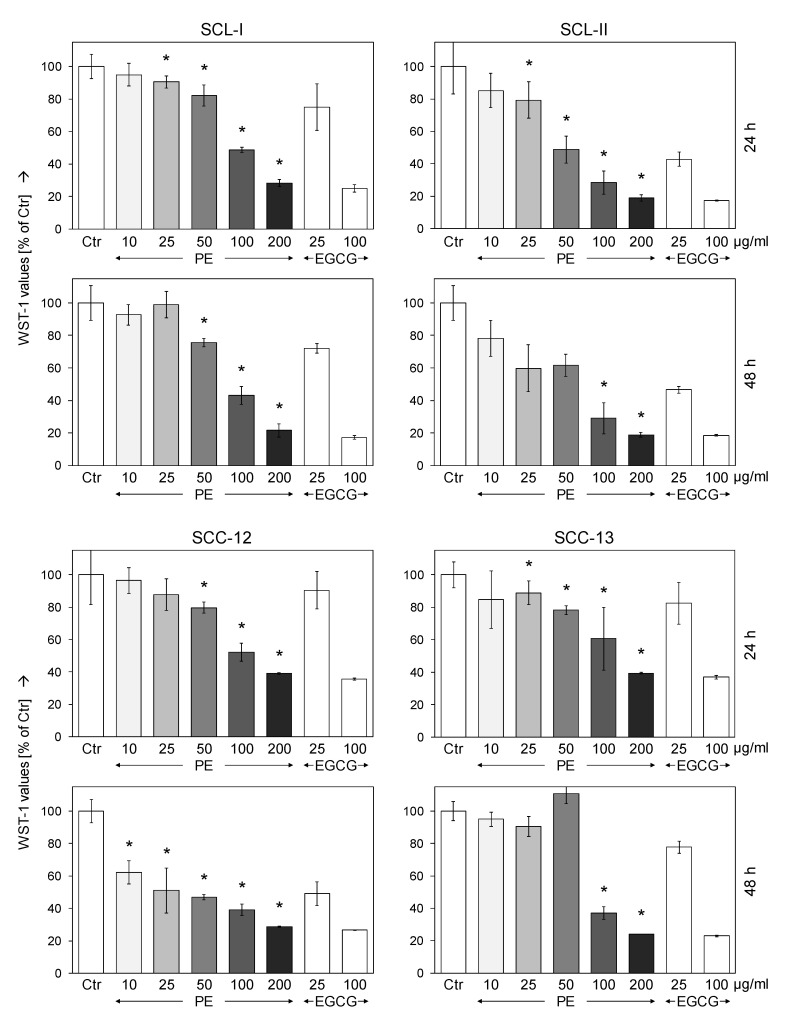
**Reduced cell proliferation by PE.** SCL-I, SCL-II, SCC-12, and SCC-13 were seeded in 96-well pates and were treated with increasing concentrations of PE (10 - 200 µg/mL). As controls, EGCG was used at 25 and 100 µg/mL. Cell proliferation was quantified by WST-1 assay at 24 h and at 48 h of treatment. One of two independent experiments is shown here, each one consisting of triplicate values. Effects on cell proliferation are shown as percentages of non-treated controls (Ctr = 100%). Statistical significance of PE treatments was calculated from all individual values (#6) and is indicated by asterisks (*p* < 0.05, as compared to Ctr).

The targeting of cancer cell proliferation and reduction of cell numbers represent important goals in anticancer therapy. Effects on cell proliferation were investigated in cSCC cell lines SCL-I, SCL-II, SCC-12, and SCC-13 in response to increasing concentrations of PE (10, 25, 50, 100 and 200 µg/mL). As determined by quantitative WST-1 assays, PE resulted in strong and dose-dependent decrease in cell counts in all four cell lines. Effects were somewhat stronger at 48 h as compared to 24 h. Thus at 48 h, cell proliferation was decreased by 100/200 µg/mL PE to 43%/22% (SCL-I), 29%/19% (SCL-II), 39%/29% (SCC-12) and to 37%/24% (SCC-13), respectively (Figure 1). For comparison, cells were treated with 25 and 100 µg/mL EGCG. Largely comparable effects were obtained as for 50 and for 200 µg/mL of PE, respectively, reflecting the roughly 55% of EGCG in PE (Figure 1). The IC50 values for PE treatment at 48 h were determined with 89 (SCL-I), 61 (SCL-II), 25 (SCC-12) and 82 µg/mL (SCC-13). For control, we investigated MRC-5 normal human fibroblasts, which were seeded and treated in an identical way as cSCC cells. In contrast to cSCC cells, no significant antiproliferative effects were found in MRC-5 at 24 h or 48 h (10–200 µg/mL PE), as determined by WST-1 assay (Supplementary Figure S1A).

### 3.2. Strongly Reduced Cell Viability and Moderate Induction of Early Apoptosis

**Figure 2:**
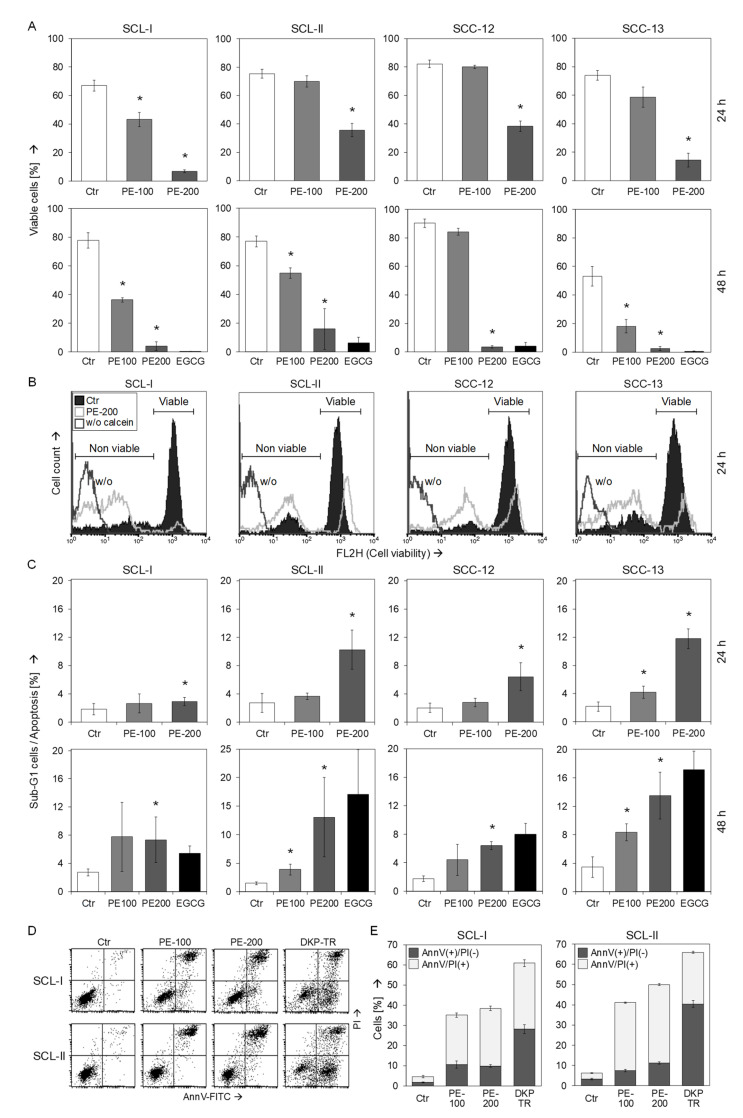
**Reduced cell viability by PE.** (**A**–**C**) SCL-I, SCL-II, SCC-12, and SCC-13 were seeded in 24-well plates and were treated with PE (100, 200 µg/mL) for 24 h and 48 h, respectively. For control, cells were also treated with 100 µg EGCG for 48 h. (**A**) Cell viability was determined by calcein-AM staining and flow cytometry. Values represent the percentage of cells with high calcein staining (= viable cells). (**B**) Examples of flow cytometry reading are shown for the cell lines at 24 h and 200 µg/mL PE treatment (overlays of treated cells vs. Ctr, logarithmic scale). Non-viable and viable cell populations are indicated. For control, cells are also shown without calcein staining (PE-200-treated, w/o). (**C**) Apoptosis was quantified by propidium iodide staining and flow cytometry (cell cycle analyses). Values represent the percentage of sub-G1 cells (=apoptotic cells). (**A**,**C**) Each one of at least two independent experiments is shown; each independent experiment consisted of triplicate values. Statistical significance for PE treatments was calculated from all individual values (#6) and is indicated by asterisks (*p* < 0.05, as compared to Ctr). (**D**) For SCL-I and SCL-II, induced cell death by PE-100 and PE-200 was determined by AnnV/PI staining. Treatment with the indirubin derivative DKP-071 in combination with TRAIL was used as control (DKP/TR). Representative flow cytometry histograms are shown of treated and control cells. (**E**) Mean values and SDs of AnnV(+)/PI(+) cells and of AnnV(+)/PI(−) cells are shown (in %). Mean values and SDs correspond to each six individual values obtained in two independent experiments with triplicates.

The effects of PE in cSCC cells were further investigated at the levels of cell viability (calcein-AM staining) and apoptosis (propidium iodide staining). Reduced cell proliferation rates, as shown above, coincided with strongly reduced cell viability at 24 h and 48 h. Thus, numbers of viable cell were reduced by PE (200 µg/mL) at 48 h to 4% (SCL-I), 16% (SCL-II), 4% (SCC-12) and 3% (SCC-13), respectively (non-treated controls: 53–90%; Figure 2A,B). In parallel with loss of cell viability, cell size somewhat decreased, as determined by reduced FSC (forward scatter) in flow cytometry. Thus, PE-200 resulted in decreased FSC of 68%, 67%, 90%, and 49%, in SCL-I, SCL-II, SCC-12, and SCC-13, respectively, as compared to controls (data not shown). The reduced size alone, however, cannot explain the strongly reduced calcein staining. Comparable loss of cell viability was obtained at 48 h with 100 µg/mL of EGCG, reflecting the 55% of EGCG in PE. MRC-5 normal human fibroblasts were again seeded and treated in an identical way as cSCC cells and were used as control. In contrast to cSCC cells, no significant loss of cell viability was found in MRC-5 at 24 h or 48 h (50–200 µg/mL PE), as determined by calcein staining (Supplementary Figure S1B).

In contrast to strongly reduced cell viability, effects on apoptosis induction, as determined by cell cycle analysis (sub-G1 cell populations), remained on a lower level reaching at maximum 13% +/− 7% in SCL-II and 14% +/− 4% in SCC-13 (48 h, PE-200; Figure 2C). In SCL-I and SCL-II, cell death was also investigated at 24 h by Annexin V-FITC / PI staining (AnnV/PI). As positive controls for apoptosis induction, cells were treated with a combination of an indirubin derivative (DKP-071) and TRAIL, as reported previously [24]. Early apoptotic cells were identified as AnnV(+)/PI(−) (Figure 2D). While early apoptosis was strongly enhanced by DKP/TRAIL in both cell lines (28%, 40%), PE-induced early apoptosis remained on a lower level (7–11%, Figure 2E), comparable to the values obtained by cell cycle analysis, shown above. On the other hand, numbers of AnnV(+)/PI(+) cells (late apoptosis or necrosis) were strongly enhanced at 24 h in SCL-I (29%) and SCL-II (39%; Figure 2D,E).

### 3.3. Loss of Mitochondrial Membrane Potential

**Figure 3:**
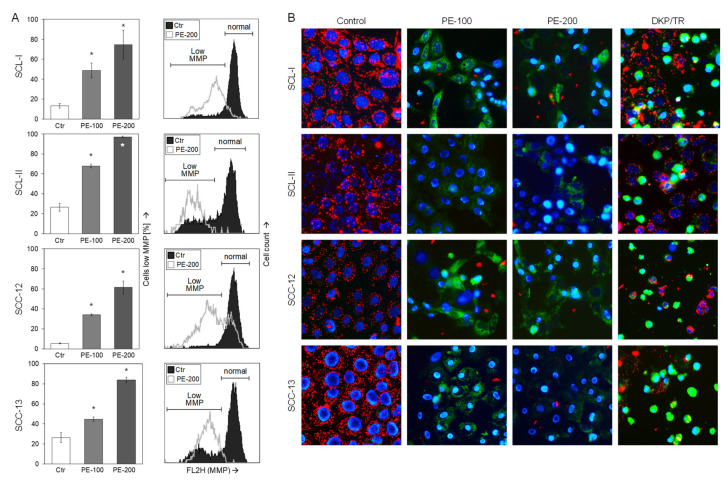
**Loss of mitochondrial membrane potential.** (**A**) SCL-I, SCL-II, SCC-12, and SCC-13 were seeded in 24-well pates and were treated with PE (100, 200 µg/mL) for 24 h. Mitochondrial membrane potential (MMP) was determined at 24 h by TMRM^+^ staining and flow cytometry. Values represent the percentage of cells with low MMP. Right, examples of flow cytometry reading are shown for 200 µg/mL treatment (overlays of treated cells vs. Ctr). Cell populations with low and normal MMP are indicated. One of two independent experiments is shown; each individual experiment consisted of triplicate values. Statistical significance was calculated from all individual values (#6) and is indicated by asterisks (*p* < 0.05, as compared to Ctr). (**B**) Cells were treated with PE-100 and PE-200 as well as with DKP-071 (10 µM)in combination with TRAIL (DKP/TR, positive control). For microscopic visualization of low MMP, cells were stained with JC-1 and counterstained with Hoechst-33342 at 24 h of treatment. Blue, nuclear staining; red, mitochondria with high (normal) MMP; faint green, JC-1-stained cytosol; bright green or turquoise, rounded and detached cells.

Maintenance of mitochondrial membrane potential (MMP) represents a critical issue in viable cells, and its early loss may be a characteristic feature of activation of intrinsic, proapoptotic pathways. We thus monitored MMP in response to PE at 24 h by applying TMRM^+^ staining and flow cytometry. In contrast to only moderately induced early apoptosis (Figure 2C,E), loss of MMP at 24 h appeared as a strong effect. As seen in flow cytometry charts, almost the whole cell populations of SCL-I, SCL-II, SCC-12, and SCC-13 were shifted, resulting in values of 75%, 97%, 61%, and 84% of cells with low MMP (200 µg/mL PE; Figure 3A).

Loss of MMP in PE-treated cells was further visualized by JC-1/ Hoechst-33342 double staining. While cell nuclei are stained blue with Hoechst-33342, the cationic dye JC-1 accumulates in mitochondria of viable cells, where it forms red fluorescent aggregates. Upon loss of MMP, however, JC-1 locates to the cytosol and fluorescence shifts from red to green. Treatment with an indirubin derivative (DKP-071, 10 µM) in combination with TRAIL was applied as positive control, as reported previously [24]. Whereas vital control cells were characterized by red-stained mitochondria, PE-100 and PE-200 treatment resulted in complete loss of red mitochondrial staining, reflecting loss of MMP. Only blue nuclear staining and green cytosolic staining remained, due to fact that cytosolic JC-1 is green (Figure 3B). However, upon PE treatment, there was only little incidence of a typical apoptotic cell morphology characterized by rounding and detachment of cells as well as by membrane blebbing, which was typically seen in positive control cells (DKP-071/TRAIL, Figure 3B). Thus, loss of MMP by PE appeared as a strong effect but was decoupled from apoptosis induction.

### 3.4. Caspase Activation and Upregulation of p21

**Figure 4:**
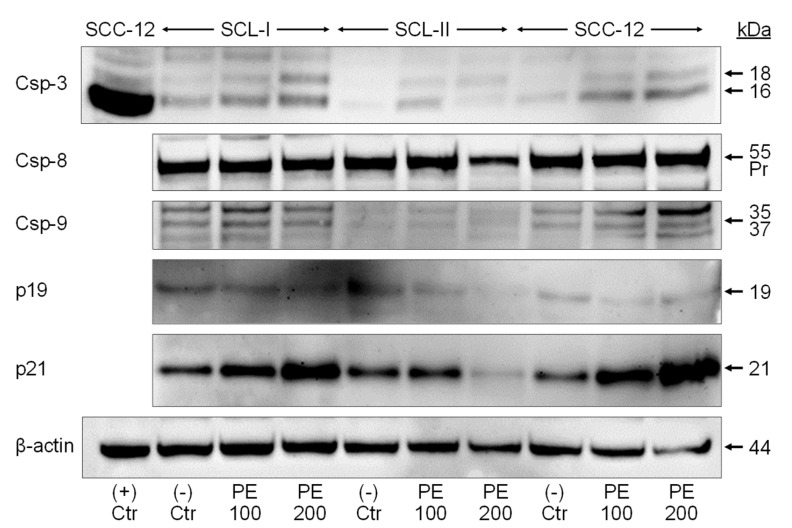
**Weak caspase activation.** Expression of caspase-3, caspase-8, caspase-9, p19, and p21 is shown by Western blotting in cell lines SCL-I, SCL-II, and SCC-12. Cells were treated for 24 h with PE (100, 200 µg/mL). Protein size (in kDa) is indicated on the right side, as determined in comparison to a protein size marker separated in parallel. Caspase activation is seen either by characteristic cleavage products, as 18/16 kDa for caspase-3 and 35/37 kDa for caspase-9 or by loss of the caspase-8 proform (55 kDa). For demonstrating full caspase-3 activation, a positive control is shown, (+)Ctr, consisting of SCC-12 cells treated with an indirubin derivative in combination with the death ligand TRAIL (TNF-related apoptosis-inducing ligand [24]. Expression of β-actin is shown as loading control. Largely similar results were obtained in two independent Western blot experiments using independent series of cell extracts.

To identify protein factors that may mediate apoptosis induction as well as inhibition of cell proliferation and cell viability by PE, the cell cycle inhibitors p19 and p21, the main effector caspase-3 as well as proapoptotic initiator caspases (−8 and −9) were investigated by Western blotting (Figure 4). Some induction of caspase-3 processing was seen in response to PE treatment (characteristic cleavage products of 18 and 16 kDa), indicative of caspase-3 activation. However, this remained on a relatively low level, as seen in comparison with a positive control consisting of SCC-12 cells treated with the indirubin derivative DKP-071 in combination with TRAIL [24]. Semi-quantitative analyses revealed induction factors of 3x − 4x for the active p16 caspase-3 cleavage product after PE treatment, while p16 was induced > 60-fold in the positive control. Similarly, only weak activation was seen for initiator caspases of the proapoptotic extrinsic and intrinsic pathways. Thus, caspase-8 proform was downregulated to 64% in SCL-II by 200 mg/mL PE, and caspase-9 cleavage products (35/37 kDa) were slightly upregulated by 200 µg/mL PE in SCL-I (1.7x) and in SCC-12 (3.7x). The weak caspase processing was in agreement with only moderately induced apoptosis described above.

As concerning inhibition of cell proliferation, we found dose-dependent upregulation of the CDK inhibitor p21 (21 kDa) by PE-200 in two cell lines (SCL-I, 2.9-fold; SCC-12, 3.1-fold), whereas there was no upregulation in SCL-II. Also, a representative of the INK4A family, p19, was not upregulated (Figure 4).

### 3.5. No Dependence on Caspases or the Proapoptotic Bcl-2 Proteins Bax and Bak

**Figure 5:**
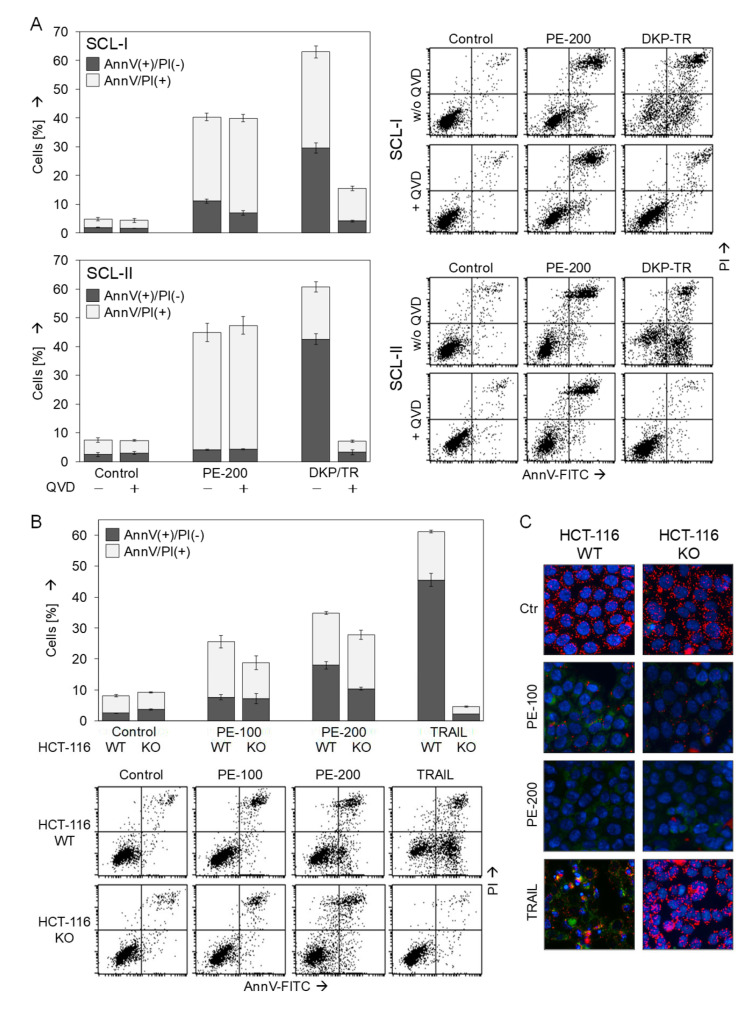
**Less effects of caspase inhibition and Bax/Bak knockdown.** (**A**) SCL-I and SCL-II cells were treated with 200 µg/mL PE (PE-200) or with an indirubin derivative DKP-071 in combination with TRAIL (DKP/TR, positive control). In addition, cells received the pancaspase inhibitor Q-VD-Oph (10 µM), when indicated. (**B**,**C**) HCT-116 WT cells and HCT-116 double knockout cells for Bax and Bak (KO) were treated with PE (100 or 200 µg/mL) or with TRAIL (positive control). (**A**,**B**) Cell death analysis by AnnV/PI staining and flow cytometry was performed after 24 h. Representative flow cytometry histograms of treated and control cells are shown on the right side or below. Mean values and SDs of two cell death fractions, namely AnnV(+)/PI(−) and AnnV(+)/PI(+) cells are shown (in %). (**C**) For microscopic visualization of low MMP and of morphological changes, cells were double stained with JC-1/Hoechst-33342 at 24 h. Blue, nuclear staining; red, mitochondria with high (normal) MMP; faint green, JC-1-stained cytosol; bright green or turquoise, rounded and detached cells.

As seen in Figure 2D,E, PE treatment resulted in less increase in early apoptotic cells characterized by AnnV(+)/PI(−), whereas numbers of late apoptotic/necrotic cells characterized by AnnV(+)/PI(+) were strongly increased. To further prove, whether cells may die by apoptosis, i.e., via a caspase-dependent pathway, and to evaluate the possible significance of caspases in PE-induced cell death, the pan-caspase inhibitor Q-VD-Oph was applied in SCL-I and SCL-II, and cell death was evaluated by AnnV/PI staining. When considering AnnV(+)/PI(−) cells (early apoptosis), the applied positive control (DKP-071/TRAIL) induced apoptosis in 30% and 43% of SCL-I and SCL-II cells, respectively. This proapoptotic effect was completely prevented by 10 µM Q-VD-Oph (Figure 5A). Similarly, induction of AnnV(+)/PI(+) cells by DKP-071/TRAIL was strongly reduced in SCL-I and was prevented in SCL-II by Q-VD-Oph, indicating late apoptotic (secondary necrotic) cells. In contrast, cell death induced by PE-200 was not significantly decreased by Q-VD-Oph, indicating also caspase-independent and non-apoptotic pathways induced by PE.

Mitochondrial apoptosis is typically mediated by either one of the multi-domain Bcl-2 proteins Bax or Bak [17]. Their possible role in PE-induced cell death was evaluated in a cell culture model based on HCT-116 colon carcinoma cells. While HCT-116 WT cells express functional Bax and Bak, the double knockout strain is deficient for both proteins (HCT-116 KO), as described by Wang and Joule [19]. In our experiments, HCT-116 WT cells were sensitive for TRAIL (46% apoptosis) as well as showed a comparable response to PE treatment at 24 h as cSCC cells. Indicating the important roles of Bax/Bak in these cells, TRAIL-induced apoptosis was completely abolished in HCT-116 KO cells, whereas PE-mediated effects were much less decreased in KO cells (Figure 5B). When considering both AnnV(+)/PI(−) and AnnV(+)/PI(+) cells, the effects of PE-100 decreased from 26% to 19%, and the effects of PE-200 decreased from 35% to 28%.

To further analyze the possible role of Bax/Bak in PE-induced loss of MMP, HCT-116 WT and KO cells were stained with JC-1/Hoechst-33342. Again, the effects of TRAIL, here loss of MMP, were completely blocked in Bax/Bak-deficient cells, whereas loss of MMP by PE was less affected (Figure 5C). Together, these data indicate that also Bax and Bak were not essentially required for PE-induced cell death and loss of MMP, again suggesting the additional role of non-apoptotic, alternative pathways involved in PE-induced cell death.

### 3.6. Role of Reactive Oxygen Species

Increasing evidence in recent years has shown that the control of reactive oxygen species may play vital roles in cellular homeostasis, and induced ROS production can trigger apoptosis programs in cancer cells. On the other hand, antioxidative activities have been described for EGCG, a major constituent of PE [9]. Thus, the control of ROS in response to PE appeared of particular interest.

**Figure 6:**
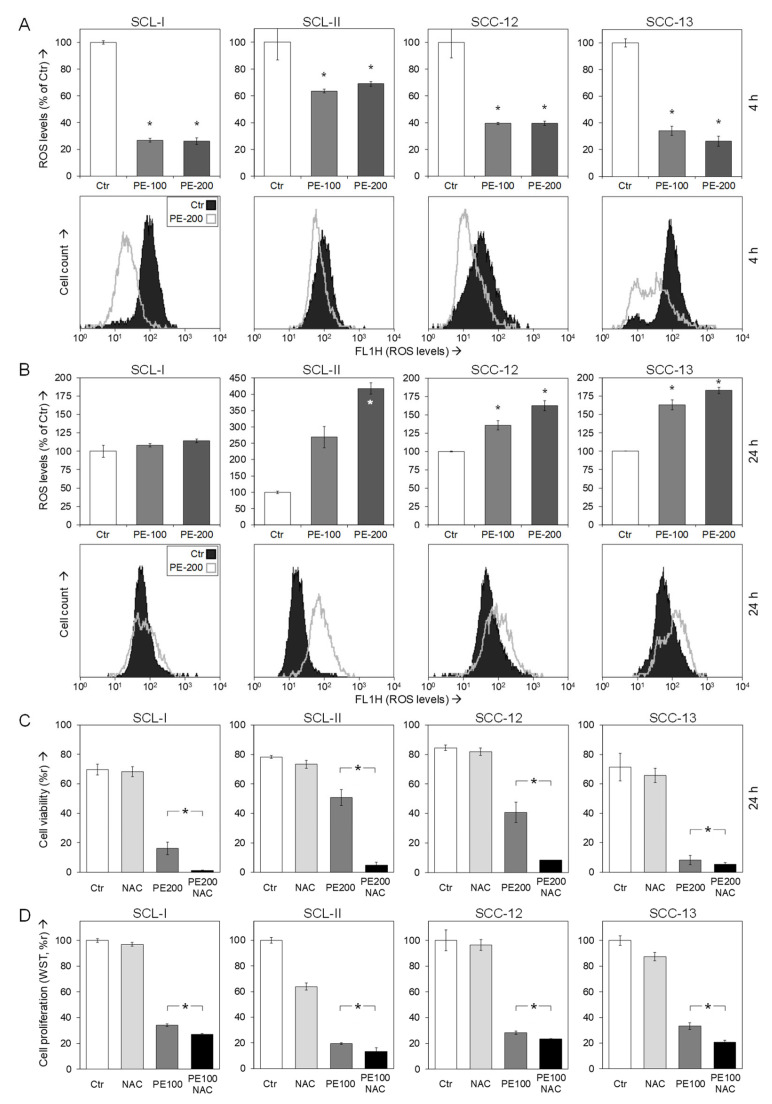
**Dysregulation of ROS by PE.** (**A**,**B**) SCC cells were seeded in 24-well plates and treated with PE (100, 200 µg/mL) for 4 h (**A**) and for 24 h (**B**), respectively. Cellular levels of ROS were determined by H_2_DCF-DA staining and flow cytometry. Values represent means of ROS levels in cells in percent, as compared to non-treated controls (Ctr, 100%). Examples of flow cytometry readings are shown below (overlays of cells treated with 200 µg/mL PE vs. Ctr). Each one of two independent experiments is shown; each individual experiment consisted of triplicate values. Statistical significance was calculated from all individual values (#6) and is indicated by asterisks (*p* < 0.05, as compared to Ctr). (**C**) SCC cell lines seeded in 24-well plates were treated with PE (200 µg/mL) +/− N-acetylcysteine (NAC, 1 mM), as indicated. Cell viability was determined at 24 h of treatment by calcein-AM staining and flow cytometry. Values represent the percentage of cells with high calcein staining (viable cells). (**D**) SCC cell lines seeded in 96-well plates (10,000 cells/well) were treated with PE (100 µg/mL) +/− 1 mM NAC, as indicated. Cell proliferation rates were determined at 24 h of treatment by WST-1 assay. (**C**,**D**) At least two independent experiments revealed highly comparable results; each individual experiment consisted of triplicate values. Statistical significance of differences between PE/NAC-treated cells and PE-treated cells was calculated from all individual values in a group (at least 6) and is indicated by asterisks (*p* < 0.05).

Indeed, reduced ROS levels were obtained in all four cell lines as an early response to PE treatment (100/200 µg/mL), as determined by the ROS-sensitive dye H_2_DCF-DA and flow cytometry. Thus, ROS were reduced in response to 200 µg/mL at 4 h to 26% (SCL-I), 69% (SCL-II), 40% (SCC-12) and 26% (SCC-13), respectively. As seen from the cytometry charts, the whole cell populations appeared as responsive (Figure 6A).

The situation, however, reversed at 24 h. Then, ROS levels were enhanced in response to PE. Values of 420% (SCL-II), 160% (SCC-12) and 180% (SCC-13) were determined for 200 µg/mL PE, and again almost all cells appeared as responsive, as seen in the cytometry charts (Figure 6B). In SCL-I, the early reduction of ROS was also abolished, but its induction at 24 h was not significant (114%).

In order to assess the relations between ROS and the observed loss of cell viability in response to PE, we used the antioxidant N-acetyl cysteine (NAC, 1 mM). While NAC itself remained almost without effect on cell viability, it significantly further enhanced the reduction of cell viability by PE. Thus, viability values at 24 h decreased from 16% to 1% (SCL-I), from 51% to 5% (SCL-II), from 41% to 9% (SCC-12) and from 8% to 6% (SCC-13), respectively (Figure 6C). A similar tendency was seen for the WST-1 values at 24 h, when cells were treated with a combination of PE-100 and NAC (1 mM), as compared to PE treatment alone. Thus, WST-1 values decreased from 34% to 27% (SCL-I), 20% to 13% (SCL-II), 28% to 23% (SCC-12) and from 33% to 21% (SCC-13) (Figure 6D). These data suggest that the PE-mediated ROS imbalance may be the signal for its inhibitory effects.

## 4. Discussion

Due to high incidence and malignant progression, cSCC represents a severe health problem worldwide [3,4]. Additionally, treatment of field cancerization in AK is crucial, concerning the high risk to proceed to invasive cSCC [6]. Polyphenols as green tea extracts are under basic research for their potential use as antitumor agents [28,29]. Thus, sinecatechins (PE) includes a mixture of different catechin derivatives and other green tea components as epigallocatechin-3-gallate (EGCG), epigallocatechin, epicatechin-3-gallate, epicatechin, gallocatechins, gallocatechin gallate, gallic acid, caffeine, and theobromine [9].

PE ointment has been approved in 2006 for treatment of genital warts, after extensive testing in clinical trials, which showed complete clearance in more than 50% of patients and few side effects [30,31]. Based on this experience, it was also suggested for treatment of AK [7]. Although there is a large body of literature on green tea and skin cancer, a large part of the literature deals with the protective effects of green tea extracts, e.g., against irradiation-induced carcinogenesis [32], while other papers focus on the clinical use of PE for treatment of AK [7,8]. On the other hand, there is much less information on its direct mechanisms and mode of action in skin cancer cells. We thus investigated the effects of PE in cutaneous SCC cell lines, which represent suitable models for cSCC as well as for AK, due to the definition of AK as cSCC in situ. The effects of PE may be furthermore compared to those of epigallocatechin gallate (EGCG), which represents the most abundant catechin in green tea [9], and was here applied as a positive control.

Significant antitumor effects were obtained at the levels of cSCC cell proliferation and viability. Effects were comparable to the effects of EGCG, when used at half the concentration. Considering the 55% of EGCG in sinecatechins, these findings strongly suggestthat EGCG was the active compound here. PE treatment resulted in less induction of early apoptotic cells, whereas numbers of late apoptotic/necrotic cells were strongly increased, as shown by Annexin V/PI double staining. Also in mammary carcinoma cells (MCF-7), strong antiproliferative effects (up to 90%) had been reported in response to EGCG, while apoptosis was only at 6% [10]. In contrast, more pronounced apoptosis induction by EGCG was reported in other cancer cells. Thus, in lung cancer cells (H1299, A549), apoptosis induction by EGCG was up to 40% [11]. In gastric cancer cells (SGC7901), inhibition of cell proliferation was up to 70-fold, and apoptosis was up to 25% [12]. In ER-negative breast cancer cells (MDA-MB-468), EGCG resulted in strongly inhibited cell proliferation, loss of cell viability (up to 80%) as well as induced apoptosis (up to 70%) [33]. Furthermore, in colorectal cancer cells (SW480, SW620, LS411N), EGCG resulted in significant inhibition of cell proliferation and induction of apoptosis (up to 60%) [34]. Thus, while green tea components strongly induced apoptosis in other cell types, this effect could be masked in cSCC cells.

When raising the question of possible effects in normal cells, which may result in side effects in the clinical setting, it may be considered that green tea extracts have been used in traditional Chinese medicine for many years and PE topical treatment has been tested in several clinical trials. Major side effects are not known and have not been reported from the clinical trials. Thus, polyphenon E was approved for treatment of genital warts [30,31]. Here, the effects of PE were investigated in normal human fibroblasts, which did not show significant inhibition at the levels of cell viability and cell proliferation. Comparably, almost no inhibitory effects were reported for PE in combination with lactoferrin in normal human gingival fibroblast cells [35]. Additionally, EGCG and PE preferentially inhibited growth of colon cancer cells when compared with a normal human fetal colon cell line [36].

Inhibition of cell proliferation by PE correlated with dose-dependent upregulation of the cyclin-dependent kinase (CDK) inhibitor p21^Cip1^ in two of three investigated cSCC cell lines. This inhibitor mainly targets CDK2, although capable of inhibiting also other cyclin/CDK complexes [37,38]. Besides p21, the family of Cip/Kip inhibitors includes also p27 and p57 [14]. Upregulation of p21 and p27 in response to EGCG has also been reported in MCF-7 breast carcinoma cells, in prostate carcinoma cells as well as in SCC-13 [39,40,41]. These CDK inhibitors may thus contribute to the antiproliferative effects of EGCG/PE, but the lack of p21 upregulation in SCL-II and its only limited upregulation in SCL-I and SCC-12 may suggest that this factor cannot be solely responsible for the antiproliferative effects induced by PE. Inhibition of cell proliferation may also be related to targeting EGFR and Notch pathways, as shown in SCC cells [42]. It is well known that the regulation of cell proliferation and viability is due to multiple factors and multiple pathways.

Concerning the mechanisms of PE-induced cell death in cSCC cells, loss of mitochondrial membrane potential (MMP), caspase activation and the roles of Bax and Bak were investigated. Significant caspase-3 activation in response to EGCG had been reported in bladder and colon cancer cells [43,44]. In cSCC cells, however, activation/processing of initiator caspases (-8 and -9) and the major effector caspase-3 remained on a lower level. Consistent with this, PE-mediated cell death was less affected by a pancaspase inhibitor (Q-VD-Oph), which, on the other hand, completely abolished apoptosis induced by an indirubin derivative in combination with TRAIL [24], used a positive control. This clearly indicated that also caspase-independent, non-apoptotic pathways were induced by PE in cSCC cells, besides some early induction of apoptosis.

Early loss of MMP often correlates with release of proapoptotic mitochondrial factors, e.g., cytochrome c, which then trigger intrinsic proapoptotic pathways via caspase-9/caspase-3 [17,18]. Thus, in HeLa cervical cancer cells, loss of MMP by EGCG was reported at 24 h [45,46]. Interestingly, PE resulted in a complete loss of MMP at 24 h in cSCC cells. However, there was only little incidence of typical apoptotic cell morphology upon PE treatment. Thus, loss of MMP by PE appeared as a strong effect but was decoupled from apoptosis induction.

Activation of intrinsic proapoptotic pathways typically depends on the multidomain proapoptotic proteins Bax and/or Bak [17]. Their possible role in PE-mediated cell death was evaluated in a cell culture model based on HCT-116 colon carcinoma cells [19,20,21], which showed a comparable response to PE as cSCC cells in Annexin V/PI staining as well as in JC-1 staining. While TRAIL-induced apoptosis was completely abolished in HCT-116 Bax/Bak double knockout cells, PE-mediated effects were less decreased. Together, these data indicate that neither caspases nor Bax or Bak were essentially required for the main effects of PE on cell death and loss of MMP. This was clearly suggestive for additional, non-apoptotic, alternative pathways involved in PE-induced cell death.

The intracellular formation of different kinds of reactive oxygen species can result in molecular damage and increased oxidative activity, and ROS are involved in multiple signaling pathways, including cell death signaling pathways. For different kinds of disease as of the neuronal, cardiovascular and nervous systems as well as in aging, ROS play important roles [22]. For polyphenols as for EGCG, antioxidative and thus protective effects have been reported, e.g., in cardiovascular disease, Alzheimer’s disease and in skin damage [47,48,49].

On the other hand, reactive oxygen species may critically contribute to the induction of apoptosis and other cell death pathways in skin cancer cells, as shown for an iron-substituted nucleoside analogue in melanoma cells [50], for celecoxib in cSCC cells [23] and for indirubin derivatives in cutaneous T-cell lymphoma, melanoma and cSCC cells [24,51,52]. ROS may derive from mitochondrial leakage or other sources [53], but their relations to apoptosis pathways are still largely elusive to date. ROS production was also reported in different kinds of cSCC therapy, such as chemotherapy, photothermal, and photodynamic therapy [54,55,56].

While for other treatments the effects on ROS were rapid and strong upregulation, the effects of PE in cSCC cells were biphasic. Thus, PE resulted in an initial decrease in ROS (4 h) followed by a moderate ROS upregulation at 24 h. Increased ROS levels in response to EGCG had also been reported in HepG2 hepatocellular carcinoma cells [57], in primary effusion lymphoma cells [58] and in malignant mesothelioma cells [59]. In all three reports, ROS induction was also obtained at later times, namely 24 h.

Thus, polyphenols may exert both antioxidative and pro-oxidative activities. Ambivalent effects on ROS levels have also been reported for other agents. Thus, the antioxidant vitamin C may also reveal pro-oxidative activities in cancer cells [60]. Pro- and antioxidant effects have also been reported for flavonoids in plant extracts [61] and for different phenolic compounds in food [62]. The modulation of ROS appears as a suitable strategy for selective targeting cancer cells. Thus, cancer cells are often characterized by elevated ROS levels and may on one hand be more sensitive for ROS-inducing agents. On the other hand, cancer cells are adjusted to the elevated ROS levels and may thus be also more sensitive to a suppression of ROS, as shown here for PE. A switch from antioxidative to pro-oxidative activity may depend on the cellular redox systems, ROS balance by pro- and antioxidative enzymes, pH, iron and oxygen [63].

We suggest, that PE mediates an imbalance of ROS in cSCC cells, characterized by an initial decrease and subsequent increase. This may be responsible for the decreased cell viability observed in course of PE treatment in cSCC cells. The hypothesis was supported by the use of the additional antioxidant N-acetylcysteine (NAC). While NAC alone remained without significant effects on cSCC viability, the combination of PE with NAC resulted in a further strong decrease in cell viability. Of course, early antioxidative effects cannot be a sufficient step for decreasing cell viability. The green tea extract PE may have even more activities beyond its antioxidative effects that finally lead to the inhibition of cSCC cells.

## 5. Conclusions

Sinecatechins has been approved in 2006 as ointment for treatment of genital warts (Polyphenon E, Veregen). Based on these experiences, it was also suggested for treatment of actinic keratosis, however, mechanistic studies in skin cancer cells were rare. In this investigation, we demonstrate the particular high activity of sinecatechins in cSCC cells, which also serve as models for actinic keratosis. Induced cell death was, however, less related to classical apoptosis pathways, e.g., activation of caspases or of Bax and Bak. Rather, alternative cell death pathways were also involved, which enclose loss of MMP and a dysbalance of ROS, finally leading to loss of cell viability and cell proliferation. These data may support ideas for further development of PE in the treatment of AK. In general, the considering of targeted strategies for ROS may extend the therapeutic options in cancer therapy.

## Data Availability

Data is contained within the manuscript and Supplementary Materials.

## Supplementary Materials

The following supporting information can be downloaded at: https://www.mdpi.com/article/10.3390/antiox11071416/s1, Figure S1: MRC-5 cells (human fetal lung fibroblast cells) were seeded and treated in an identical way as cSCC cells. Cell proliferation (A), WST-1 assay) and cell viability (B), calcein staining) were performed at 24 h and at 48 h, respectively. Mean values and SDs were calculated for 6 (A) and 4 (B) individual wells.
