# Supplementary material for: Inhibition of Cell Proliferation and Cell Viability by Sinecatechins in Cutaneous SCC Cells Is Related to an Imbalance of ROS and Loss of Mitochondrial Membrane Potential
Source: Antioxidants (Basel). 2022 Jul 21;11(7):1416. doi: 10.3390/antiox11071416 (PMC9312260; doi:10.3390/antiox11071416)
Supplement: Supplementary file 1 [file antioxidants-11-01416-s001.zip › antioxidants-1803095-supplementary.pdf]

antioxidants

Supplementary Materials: Inhibition of Cell Proliferation and Cell Viability by Sinecatechins in Cutaneous SCC Cells Is Related to an Imbalance of ROS and Loss of Mitochondrial Membrane Potential

Jiaqi Zhu [1,2], Bernd Gillissen [3], Dieu Linh Dang Tran [1,4], Stefanie May [1], Claas Ulrich [1], Eggert Stockfleth [5] and Jürgen Eberle [1,*]

[1] Skin Cancer Centre Charité, Department of Dermatology and Allergy, Charité–Universitätsmedizin Berlin, Charitéplatz 1, 10117 Berlin, Germany; jiaqi.zhu@charite.de (J.Z.); tran-dieu-linh.dang@charite.de (D.L.D.T.); stefanie.may@charite.de (S.M.); claas.ulrich@charite.de (C.U.)
[2] Department of Gynecology and Obstetrics, Jilin University, 130001 Changchun, China
[3] Department of Hematology, Oncology, and Tumor Immunology, Charité–Universitätsmedizin Berlin, 13125 Berlin, Germany; bernhard.gillissen@charite.de
[4] Beuth-Hochschule für Technik Berlin–University of Applied Sciences, Luxemburger Str. 10, 13353 Berlin, Germany
[5] Dermatologie, Venerologie und Allergologie, Klinikum Bochum, Ruhr-Universität Bochum, Gudrunstr. 56, 44791 Bochum, Germany; e.stockfleth@klinikum-bochum.de
* Correspondence: juergen.eberle@charite.de; Tel.: +49-30-450-518-383

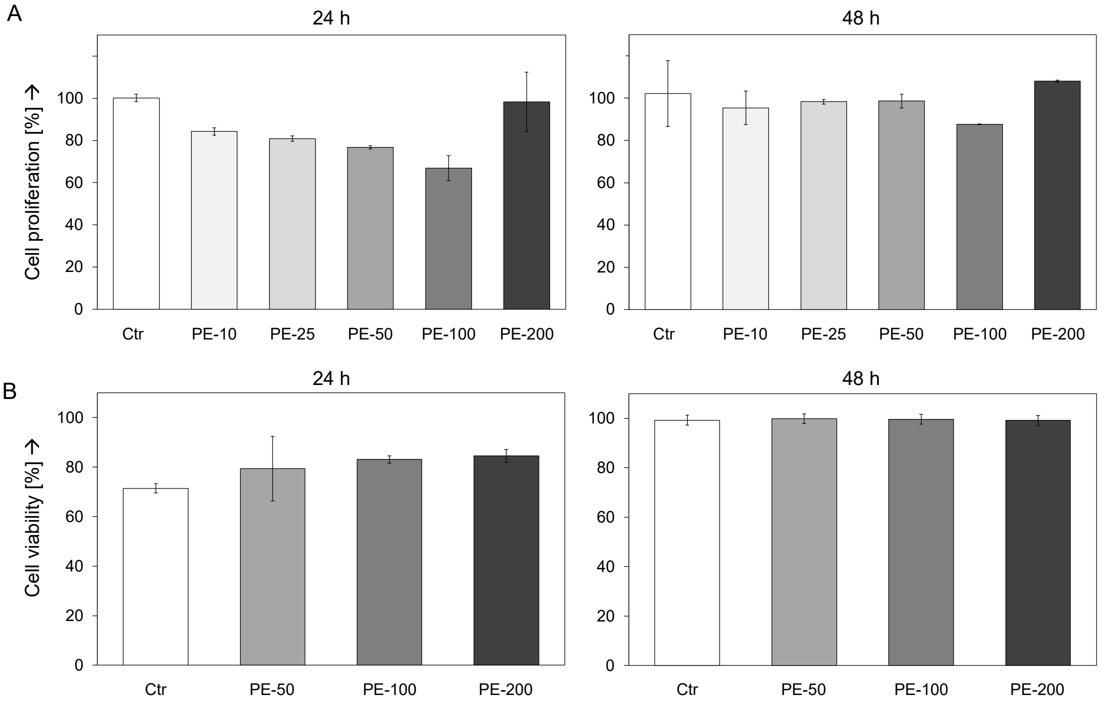

Figure S1. MRC-5 cells (human fetal lung fibroblast cells) were seeded and treated in an identical way as cSCC cells. Cell proliferation (A, WST-1 assay) and cell viability (B, calcein staining) were performed at 24 h and at 48 h, respectively. Mean values and SDs were calculated for 6 (A) and 4 (B) individual wells.
